# Supplementary material for: Quantifying contact patterns in response to COVID-19 public health measures in Canada
Source: BMC Public Health. 2021 Nov 8;21:2040. doi: 10.1186/s12889-021-12080-1 (PMC8574152; doi:10.1186/s12889-021-12080-1)
Supplement: Supplementary file 2 — Additional file 2. [file 12889_2021_12080_MOESM2_ESM.docx]

**Additional File 2: Contact Matrices for Figure 3.**

**All Tables**: Average number of contacts per 24 hour period for all reported contacts and those stratified by setting in which the contact occurred for each wave of the survey. Settings included all contacts, contacts made at home, contacts made in the workplace, contacts made at school, and contacts made everywhere else (including social contacts). The number of contacts in the September and December surveys were truncated at 75 contacts per respondent. Data were weighted on age and household size. The 2016 Canadian census was used for demographics in correcting for probability of contact within the population. Missing contact age was sampled from age-matched participants’ contacts.

**Table B1.** May 2020 – All Contacts

|  |  | **Contact Age Group** | | | | | | | |
| --- | --- | --- | --- | --- | --- | --- | --- | --- | --- |
|  |  | **0-4 years** | **5-17 years** | **18-29 years** | **30-39 years** | **40-49 years** | **50-59 years** | **60-69 years** | **70+ years** |
| **Participant Age Group** | **0-4 years** | *NA* | *NA* | *NA* | *NA* | *NA* | *NA* | *NA* | *NA* |
|  | **5-17 years** | *NA* | *NA* | *NA* | *NA* | *NA* | *NA* | *NA* | *NA* |
|  | **18-29 years** | 0.11010363 | 0.27331606 | 0.9455959 | 0.3458549 | 0.3056995 | 0.4235751 | 0.1580311 | 0.07253886 |
|  | **30-39 years** | 0.16720955 | 0.30836048 | 0.3550489 | 0.6927253 | 0.3083605 | 0.2290988 | 0.2204126 | 0.09337676 |
|  | **40-49 years** | 0.05534591 | 0.50062893 | 0.3773585 | 0.3169811 | 0.5383648 | 0.3345912 | 0.1421384 | 0.15849057 |
|  | **50-59 years** | 0.02850356 | 0.18764846 | 0.4239905 | 0.2470309 | 0.2992874 | 0.5320665 | 0.2066508 | 0.17458432 |
|  | **60-69 years** | 0.04776739 | 0.08930426 | 0.2523364 | 0.3364486 | 0.2242991 | 0.2481828 | 0.4527518 | 0.20560748 |
|  | **70+ years** | 0.01910828 | 0.12261146 | 0.1910828 | 0.1847134 | 0.2643312 | 0.1926752 | 0.2452229 | 0.47929936 |

**Table B2.** May 2020 – Home

|  |  | **Contact Age Group** | | | | | | | |
| --- | --- | --- | --- | --- | --- | --- | --- | --- | --- |
|  |  | **0-4 years** | **5-17 years** | **18-29 years** | **30-39 years** | **40-49 years** | **50-59 years** | **60-69 years** | **70+ years** |
| **Participant Age Group** | **0-4 years** | *NA* | *NA* | *NA* | *NA* | *NA* | *NA* | *NA* | *NA* |
|  | **5-17 years** | *NA* | *NA* | *NA* | *NA* | *NA* | *NA* | *NA* | *NA* |
|  | **18-29 years** | 0.07564103 | 0.17179487 | 0.46794872 | 0.12948718 | 0.14615385 | 0.27179487 | 0.09358974 | 0.02564103 |
|  | **30-39 years** | 0.14469453 | 0.25294748 | 0.10610932 | 0.38799571 | 0.07824223 | 0.05251876 | 0.10825295 | 0.04715970 |
|  | **40-49 years** | 0.04500000 | 0.45750000 | 0.15000000 | 0.10875000 | 0.31500000 | 0.11625000 | 0.04875000 | 0.07000000 |
|  | **50-59 years** | 0.01180638 | 0.16292798 | 0.23730815 | 0.05667060 | 0.09327037 | 0.30814640 | 0.10507674 | 0.07319953 |
|  | **60-69 years** | 0.02769231 | 0.05538462 | 0.10153846 | 0.15589744 | 0.07179487 | 0.11179487 | 0.33641026 | 0.11589744 |
|  | **70+ years** | 0.01415094 | 0.06761006 | 0.03616352 | 0.06603774 | 0.11320755 | 0.08176101 | 0.13836478 | 0.36792453 |

**Table B3:** May 2020 – Work

|  |  | **Contact Age Group** | | | | | | | |
| --- | --- | --- | --- | --- | --- | --- | --- | --- | --- |
|  |  | **0-4 years** | **5-17 years** | **18-29 years** | **30-39 years** | **40-49 years** | **50-59 years** | **60-69 years** | **70+ years** |
| **Participant Age Group** | **0-4 years** | *NA* | *NA* | *NA* | *NA* | *NA* | *NA* | *NA* | *NA* |
|  | **5-17 years** | *NA* | *NA* | *NA* | *NA* | *NA* | *NA* | *NA* | *NA* |
|  | **18-29 years** | 0.006418485 | 0.043645700 | 0.132220796 | 0.091142490 | 0.082156611 | 0.055198973 | 0.028241335 | 0.015404365 |
|  | **30-39 years** | 0.005347594 | 0.012834225 | 0.104812834 | 0.161497326 | 0.147593583 | 0.111229947 | 0.042780749 | 0.020320856 |
|  | **40-49 years** | 0.001246883 | 0.009975062 | 0.072319202 | 0.078553616 | 0.112219451 | 0.123441397 | 0.037406484 | 0.023690773 |
|  | **50-59 years** | 0.005903188 | 0.005903188 | 0.034238489 | 0.085005903 | 0.099173554 | 0.105076741 | 0.037780401 | 0.018890201 |
|  | **60-69 years** | 0.000000000 | 0.001025641 | 0.010256410 | 0.011282051 | 0.012307692 | 0.020512821 | 0.019487179 | 0.005128205 |
|  | **70+ years** | 0.000000000 | 0.000000000 | 0.003134796 | 0.003134796 | 0.007836991 | 0.006269592 | 0.003134796 | 0.001567398 |

**Table B4:** May 2020 – School

|  |  | **Contact Age Group** | | | | | | | |
| --- | --- | --- | --- | --- | --- | --- | --- | --- | --- |
|  |  | **0-4 years** | **5-17 years** | **18-29 years** | **30-39 years** | **40-49 years** | **50-59 years** | **60-69 years** | **70+ years** |
| **Participant Age Group** | **0-4 years** | *NA* | *NA* | *NA* | *NA* | *NA* | *NA* | *NA* | *NA* |
|  | **5-17 years** | *NA* | *NA* | *NA* | *NA* | *NA* | *NA* | *NA* | *NA* |
|  | **18-29 years** | 0.002564103 | 0.005128205 | 0.016666667 | 0.003846154 | 0.002564103 | 0.000000000 | 0.000000000 | 0.001282051 |
|  | **30-39 years** | 0.000000000 | 0.001071811 | 0.005359057 | 0.001071811 | 0.001071811 | 0.000000000 | 0.000000000 | 0.000000000 |
|  | **40-49 years** | 0.001246883 | 0.003740648 | 0.000000000 | 0.002493766 | 0.003740648 | 0.001246883 | 0.000000000 | 0.000000000 |
|  | **50-59 years** | 0.000000000 | 0.000000000 | 0.001179245 | 0.000000000 | 0.002358491 | 0.000000000 | 0.000000000 | 0.000000000 |
|  | **60-69 years** | 0.000000000 | 0.001023541 | 0.000000000 | 0.000000000 | 0.000000000 | 0.000000000 | 0.000000000 | 0.001023541 |
|  | **70+ years** | 0.000000000 | 0.000000000 | 0.000000000 | 0.000000000 | 0.000000000 | 0.000000000 | 0.001567398 | 0.000000000 |

**Table B4:** May 2020 – Other

|  |  | **Contact Age Group** | | | | | | | | |
| --- | --- | --- | --- | --- | --- | --- | --- | --- | --- | --- |
|  |  | **0-4 years** | **5-17 years** | **18-29 years** | **30-39 years** | **40-49 years** | **50-59 years** | **60-69 years** | **70+ years** |  |
| **Participant Age Group** | **0-4 years** | *NA* | *NA* | *NA* | *NA* | *NA* | *NA* | *NA* | *NA* |  |
|  | **5-17 years** | *NA* | *NA* | *NA* | *NA* | *NA* | *NA* | *NA* | *NA* |  |
|  | **18-29 years** | 0.027061856 | 0.05283505 | 0.3260309 | 0.1211340 | 0.07345361 | 0.09536082 | 0.03479381 | 0.02963918 |  |
|  | **30-39 years** | 0.017297297 | 0.03891892 | 0.1405405 | 0.1351351 | 0.08108108 | 0.06378378 | 0.07135135 | 0.02594595 |  |
|  | **40-49 years** | 0.007528231 | 0.03011292 | 0.1555834 | 0.1267252 | 0.10915935 | 0.09410289 | 0.05520703 | 0.06398996 |  |
|  | **50-59 years** | 0.010663507 | 0.02014218 | 0.1504739 | 0.1042654 | 0.10308057 | 0.11729858 | 0.06398104 | 0.08175355 |  |
|  | **60-69 years** | 0.019648397 | 0.03516029 | 0.1406412 | 0.1695967 | 0.14167528 | 0.11478800 | 0.09824199 | 0.08479835 |  |
|  | **70+ years** | 0.004769475 | 0.05405405 | 0.1510334 | 0.1144674 | 0.14149444 | 0.10333863 | 0.10015898 | 0.11128776 |  |

**Table B5:** July 2020 – All contacts

|  |  | **Contact Age Group** | | | | | | | | |
| --- | --- | --- | --- | --- | --- | --- | --- | --- | --- | --- |
|  |  | **0-4 years** | **5-17 years** | **18-29 years** | **30-39 years** | **40-49 years** | **50-59 years** | **60-69 years** | **70+ years** |  |
| **Participant Age Group** | **0-4 years** | *NA* | *NA* | *NA* | *NA* | *NA* | *NA* | *NA* | *NA* |  |
|  | **5-17 years** | *NA* | *NA* | *NA* | *NA* | *NA* | *NA* | *NA* | *NA* |  |
|  | **18-29 years** | 0.06539510 | 0.21525886 | 1.0326975 | 0.4114441 | 0.3024523 | 0.3215259 | 0.1689373 | 0.03814714 |  |
|  | **30-39 years** | 0.13859275 | 0.16844350 | 0.3539446 | 0.7164179 | 0.3155650 | 0.2558635 | 0.2174840 | 0.07889126 |  |
|  | **40-49 years** | 0.07524272 | 0.33009709 | 0.3834951 | 0.3980583 | 0.5485437 | 0.3203883 | 0.1699029 | 0.15776699 |  |
|  | **50-59 years** | 0.03564356 | 0.12277228 | 0.4396040 | 0.2732673 | 0.3287129 | 0.4811881 | 0.2495050 | 0.19009901 |  |
|  | **60-69 years** | 0.06938776 | 0.10204082 | 0.3163265 | 0.3897959 | 0.2714286 | 0.3204082 | 0.5285714 | 0.23265306 |  |
|  | **70+ years** | 0.03017241 | 0.04310345 | 0.2112069 | 0.2241379 | 0.2370690 | 0.2629310 | 0.2456897 | 0.43103448 |  |

**Table B6:** July 2020 – Home

|  |  | **Contact Age Group** | | | | | | | | |
| --- | --- | --- | --- | --- | --- | --- | --- | --- | --- | --- |
|  |  | **0-4 years** | **5-17 years** | **18-29 years** | **30-39 years** | **40-49 years** | **50-59 years** | **60-69 years** | **70+ years** |  |
| **Participant Age Group** | **0-4 years** | *NA* | *NA* | *NA* | *NA* | *NA* | *NA* | *NA* | *NA* |  |
|  | **5-17 years** | *NA* | *NA* | *NA* | *NA* | *NA* | *NA* | *NA* | *NA* |  |
|  | **18-29 years** | 0.02989130 | 0.09239130 | 0.31250000 | 0.07608696 | 0.11413043 | 0.16032609 | 0.08695652 | 0.01630435 |  |
|  | **30-39 years** | 0.09978769 | 0.10403397 | 0.07855626 | 0.24840764 | 0.08280255 | 0.08280255 | 0.06581741 | 0.02547771 |  |
|  | **40-49 years** | 0.05084746 | 0.22276029 | 0.05569007 | 0.08716707 | 0.19128329 | 0.07263923 | 0.03874092 | 0.04600484 |  |
|  | **50-59 years** | 0.01972387 | 0.07100592 | 0.15976331 | 0.06114398 | 0.08086785 | 0.21104536 | 0.08678501 | 0.08481262 |  |
|  | **60-69 years** | 0.03469388 | 0.05306122 | 0.06938776 | 0.11632653 | 0.04693878 | 0.10408163 | 0.27346939 | 0.08775510 |  |
|  | **70+ years** | 0.01287554 | 0.01716738 | 0.03862661 | 0.03433476 | 0.06437768 | 0.09012876 | 0.07725322 | 0.19313305 |  |

**Table B7:** July 2020 – Work

|  |  | **Contact Age Group** | | | | | | | |
| --- | --- | --- | --- | --- | --- | --- | --- | --- | --- |
|  |  | **0-4 years** | **5-17 years** | **18-29 years** | **30-39 years** | **40-49 years** | **50-59 years** | **60-69 years** | **70+ years** |
| **Participant Age Group** | **0-4 years** | *NA* | *NA* | *NA* | *NA* | *NA* | *NA* | *NA* | *NA* |
|  | **5-17 years** | *NA* | *NA* | *NA* | *NA* | *NA* | *NA* | *NA* | *NA* |
|  | **18-29 years** | 0.002688172 | 0.01881720 | 0.239247312 | 0.134408602 | 0.06989247 | 0.056451613 | 0.04032258 | 0.005376344 |
|  | **30-39 years** | 0.008403361 | 0.01470588 | 0.115546218 | 0.161764706 | 0.09033613 | 0.079831933 | 0.04831933 | 0.010504202 |
|  | **40-49 years** | 0.004842615 | 0.00968523 | 0.089588378 | 0.084745763 | 0.09443099 | 0.065375303 | 0.04358354 | 0.014527845 |
|  | **50-59 years** | 0.000000000 | 0.00000000 | 0.051282051 | 0.043392505 | 0.06706114 | 0.039447732 | 0.03747535 | 0.035502959 |
|  | **60-69 years** | 0.000000000 | 0.00203252 | 0.028455285 | 0.040650407 | 0.02642276 | 0.040650407 | 0.01219512 | 0.006097561 |
|  | **70+ years** | 0.000000000 | 0.00000000 | 0.004291845 | 0.004291845 | 0.01716738 | 0.008583691 | 0.00000000 | 0.000000000 |

**Table B8:** July 2020 – School

|  |  | **Contact Age Group** | | | | | | | |
| --- | --- | --- | --- | --- | --- | --- | --- | --- | --- |
|  |  | **0-4 years** | **5-17 years** | **18-29 years** | **30-39 years** | **40-49 years** | **50-59 years** | **60-69 years** | **70+ years** |
| **Participant Age Group** | **0-4 years** | *NA* | *NA* | *NA* | *NA* | *NA* | *NA* | *NA* | *NA* |
|  | **5-17 years** | *NA* | *NA* | *NA* | *NA* | *NA* | *NA* | *NA* | *NA* |
|  | **18-29 years** | 0.002688172 | 0.010752688 | 0.010752688 | 0.000000000 | 0.002688172 | 0.002688172 | 0.000000000 | 0.000000000 |
|  | **30-39 years** | 0.000000000 | 0.002100840 | 0.002100840 | 0.008403361 | 0.006302521 | 0.002100840 | 0.002100840 | 0.000000000 |
|  | **40-49 years** | 0.000000000 | 0.002421308 | 0.002421308 | 0.000000000 | 0.004842615 | 0.002421308 | 0.000000000 | 0.002421308 |
|  | **50-59 years** | 0.000000000 | 0.000000000 | 0.000000000 | 0.003944773 | 0.001972387 | 0.001972387 | 0.001972387 | 0.000000000 |
|  | **60-69 years** | 0.000000000 | 0.006097561 | 0.000000000 | 0.004065041 | 0.006097561 | 0.000000000 | 0.000000000 | 0.000000000 |
|  | **70+ years** | 0.000000000 | 0.000000000 | 0.000000000 | 0.000000000 | 0.004291845 | 0.000000000 | 0.000000000 | 0.000000000 |

**Table B9:** July 2020 – Other

|  |  | **Contact Age Group** | | | | | | | | |
| --- | --- | --- | --- | --- | --- | --- | --- | --- | --- | --- |
|  |  | **0-4 years** | **5-17 years** | **18-29 years** | **30-39 years** | **40-49 years** | **50-59 years** | **60-69 years** | **70+ years** |  |
| **Participant Age Group** | **0-4 years** | *NA* | *NA* | *NA* | *NA* | *NA* | *NA* | *NA* | *NA* |  |
|  | **5-17 years** | *NA* | *NA* | *NA* | *NA* | *NA* | *NA* | *NA* | *NA* |  |
|  | **18-29 years** | 0.02964960 | 0.09703504 | 0.4690027 | 0.1967655 | 0.1159030 | 0.09973046 | 0.04582210 | 0.01886792 |  |
|  | **30-39 years** | 0.02953586 | 0.04641350 | 0.1540084 | 0.2995781 | 0.1329114 | 0.08860759 | 0.10337553 | 0.04641350 |  |
|  | **40-49 years** | 0.01941748 | 0.10194175 | 0.2354369 | 0.2257282 | 0.2572816 | 0.18203883 | 0.09223301 | 0.09466019 |  |
|  | **50-59 years** | 0.01584158 | 0.05148515 | 0.2277228 | 0.1643564 | 0.1782178 | 0.22772277 | 0.12277228 | 0.06930693 |  |
|  | **60-69 years** | 0.03455285 | 0.04065041 | 0.2174797 | 0.2296748 | 0.1910569 | 0.17479675 | 0.24186992 | 0.13821138 |  |
|  | **70+ years** | 0.01724138 | 0.02586207 | 0.1681034 | 0.1853448 | 0.1508621 | 0.16379310 | 0.16810345 | 0.23706897 |  |

**Table B10:** September 2020 – All contacts

|  |  | **Contact Age Group** | | | | | | | | |
| --- | --- | --- | --- | --- | --- | --- | --- | --- | --- | --- |
|  |  | **0-4 years** | **5-17 years** | **18-29 years** | **30-39 years** | **40-49 years** | **50-59 years** | **60-69 years** | **70+ years** |  |
| **Participant Age Group** | **0-4 years** | *NA* | *NA* | *NA* | *NA* | *NA* | *NA* | *NA* | *NA* |  |
|  | **5-17 years** | *NA* | *NA* | *NA* | *NA* | *NA* | *NA* | *NA* | *NA* |  |
|  | **18-29 years** | 0.20486111 | 0.84722222 | 1.7638889 | 1.3854167 | 1.0590278 | 1.1562500 | 0.6006944 | 0.5590278 |  |
|  | **30-39 years** | 0.40485830 | 0.64372470 | 1.0931174 | 1.6862348 | 1.2206478 | 0.7085020 | 0.4230769 | 0.2651822 |  |
|  | **40-49 years** | 0.21603563 | 0.83518931 | 0.8730512 | 1.2138085 | 1.4966592 | 0.8017817 | 0.3474388 | 0.2071269 |  |
|  | **50-59 years** | 0.18067227 | 0.47899160 | 0.6554622 | 0.8508403 | 0.8319328 | 0.8004202 | 0.4075630 | 0.2268908 |  |
|  | **60-69 years** | 0.02892562 | 0.20661157 | 0.3760331 | 0.4276860 | 0.4607438 | 0.4318182 | 0.4979339 | 0.2747934 |  |
|  | **70+ years** | 0.02083333 | 0.08680556 | 0.1631944 | 0.2256944 | 0.3229167 | 0.2708333 | 0.3680556 | 0.4444444 |  |

**Table B11:** September 2020 – Home

|  |  | **Contact Age Group** | | | | | | | | |
| --- | --- | --- | --- | --- | --- | --- | --- | --- | --- | --- |
|  |  | **0-4 years** | **5-17 years** | **18-29 years** | **30-39 years** | **40-49 years** | **50-59 years** | **60-69 years** | **70+ years** |  |
| **Participant Age Group** | **0-4 years** | *NA* | *NA* | *NA* | *NA* | *NA* | *NA* | *NA* | *NA* |  |
|  | **5-17 years** | *NA* | *NA* | *NA* | *NA* | *NA* | *NA* | *NA* | *NA* |  |
|  | **18-29 years** | 0.06896552 | 0.06551724 | 0.24827586 | 0.07931034 | 0.05172414 | 0.08965517 | 0.07586207 | 0.01034483 |  |
|  | **30-39 years** | 0.07474747 | 0.07676768 | 0.04646465 | 0.22020202 | 0.06262626 | 0.02828283 | 0.07474747 | 0.02828283 |  |
|  | **40-49 years** | 0.04424779 | 0.21017699 | 0.08628319 | 0.06637168 | 0.17920354 | 0.07522124 | 0.04203540 | 0.06415929 |  |
|  | **50-59 years** | 0.02092050 | 0.07740586 | 0.13807531 | 0.05230126 | 0.07322176 | 0.17573222 | 0.08158996 | 0.03765690 |  |
|  | **60-69 years** | 0.01446281 | 0.02685950 | 0.05371901 | 0.07644628 | 0.03512397 | 0.09090909 | 0.19008264 | 0.07851240 |  |
|  | **70+ years** | 0.01034483 | 0.03793103 | 0.01724138 | 0.04827586 | 0.06551724 | 0.06896552 | 0.10000000 | 0.20689655 |  |

**Table B12:** September 2020 – Work

|  |  | **Contact Age Group** | | | | | | | | | |
| --- | --- | --- | --- | --- | --- | --- | --- | --- | --- | --- | --- |
|  |  | **0-4 years** | **5-17 years** | **18-29 years** | **30-39 years** | **40-49 years** | **50-59 years** | **60-69 years** | **70+ years** |  |  |
| **Participant Age Group** | **0-4 years** | *NA* | *NA* | *NA* | *NA* | *NA* | *NA* | *NA* | *NA* | |  |
|  | **5-17 years** | *NA* | *NA* | *NA* | *NA* | *NA* | *NA* | *NA* | *NA* | |  |
|  | **18-29 years** | 0.08219178 | 0.73972603 | 1.13013699 | 1.08561644 | 0.85958904 | 0.84246575 | 0.47602740 | 0.48972603 | |  |
|  | **30-39 years** | 0.29032258 | 0.50000000 | 0.85685484 | 1.19153226 | 0.96975806 | 0.54838710 | 0.26814516 | 0.22177419 | |  |
|  | **40-49 years** | 0.14476615 | 0.49888641 | 0.60801782 | 0.96436526 | 1.11358575 | 0.62583519 | 0.22494432 | 0.08908686 | |  |
|  | **50-59 years** | 0.12788260 | 0.35849057 | 0.35220126 | 0.61635220 | 0.59119497 | 0.47169811 | 0.22222222 | 0.11530398 | |  |
|  | **60-69 years** | 0.00000000 | 0.12603306 | 0.17975207 | 0.18595041 | 0.27685950 | 0.18388430 | 0.11363636 | 0.02685950 | |  |
|  | **70+ years** | 0.00000000 | 0.01724138 | 0.02413793 | 0.03103448 | 0.03103448 | 0.02413793 | 0.07931034 | 0.05172414 | |  |

**Table B13:** September 2020 – School

|  |  | **Contact Age Group** | | | | | | | |
| --- | --- | --- | --- | --- | --- | --- | --- | --- | --- |
|  |  | **0-4 years** | **5-17 years** | **18-29 years** | **30-39 years** | **40-49 years** | **50-59 years** | **60-69 years** | **70+ years** |
| **Participant Age Group** | **0-4 years** | *NA* | *NA* | *NA* | *NA* | *NA* | *NA* | *NA* | *NA* |
|  | **5-17 years** | *NA* | *NA* | *NA* | *NA* | *NA* | *NA* | *NA* | *NA* |
|  | **18-29 years** | 0.000000000 | 0.000000000 | 0.030716724 | 0.003412969 | 0.013651877 | 0.010238908 | 0.000000000 | 0 |
|  | **30-39 years** | 0.006036217 | 0.006036217 | 0.016096579 | 0.020120724 | 0.022132797 | 0.008048290 | 0.002012072 | 0 |
|  | **40-49 years** | 0.011086475 | 0.064301552 | 0.008869180 | 0.006651885 | 0.013303769 | 0.011086475 | 0.002217295 | 0 |
|  | **50-59 years** | 0.016736402 | 0.008368201 | 0.000000000 | 0.002092050 | 0.000000000 | 0.006276151 | 0.004184100 | 0 |
|  | **60-69 years** | 0.004132231 | 0.000000000 | 0.006198347 | 0.000000000 | 0.000000000 | 0.004132231 | 0.002066116 | 0 |
|  | **70+ years** | 0.000000000 | 0.000000000 | 0.000000000 | 0.000000000 | 0.003448276 | 0.000000000 | 0.000000000 | 0 |

**Table B14:** September 2020 – Other

|  |  | **Contact Age Group** | | | | | | | | |
| --- | --- | --- | --- | --- | --- | --- | --- | --- | --- | --- |
|  |  | **0-4 years** | **5-17 years** | **18-29 years** | **30-39 years** | **40-49 years** | **50-59 years** | **60-69 years** | **70+ years** |  |
| **Participant Age Group** | **0-4 years** | *NA* | *NA* | *NA* | *NA* | *NA* | *NA* | *NA* | *NA* |  |
|  | **5-17 years** | *NA* | *NA* | *NA* | *NA* | *NA* | *NA* | *NA* | *NA* |  |
|  | **18-29 years** | 0.05136986 | 0.03424658 | 0.3493151 | 0.1986301 | 0.1198630 | 0.2020548 | 0.04109589 | 0.05136986 |  |
|  | **30-39 years** | 0.03822938 | 0.05835010 | 0.1730382 | 0.2555332 | 0.1629779 | 0.1227364 | 0.07645875 | 0.01408451 |  |
|  | **40-49 years** | 0.01552106 | 0.05986696 | 0.1729490 | 0.1751663 | 0.1929047 | 0.0886918 | 0.07760532 | 0.05321508 |  |
|  | **50-59 years** | 0.01467505 | 0.03354298 | 0.1635220 | 0.1781971 | 0.1656184 | 0.1446541 | 0.09853249 | 0.07337526 |  |
|  | **60-69 years** | 0.01033058 | 0.05371901 | 0.1363636 | 0.1652893 | 0.1487603 | 0.1528926 | 0.19214876 | 0.16942149 |  |
|  | **70+ years** | 0.01041667 | 0.03472222 | 0.1284722 | 0.1493056 | 0.2256944 | 0.1840278 | 0.19097222 | 0.20138889 |  |

**Table B15:** December 2020 – All contacts

|  |  | **Contact Age Group** | | | | | | | | |
| --- | --- | --- | --- | --- | --- | --- | --- | --- | --- | --- |
|  |  | **0-4 years** | **5-17 years** | **18-29 years** | **30-39 years** | **40-49 years** | **50-59 years** | **60-69 years** | **70+ years** |  |
| **Participant Age Group** | **0-4 years** | *NA* | *NA* | *NA* | *NA* | *NA* | *NA* | *NA* | *NA* |  |
|  | **5-17 years** | *NA* | *NA* | *NA* | *NA* | *NA* | *NA* | *NA* | *NA* |  |
|  | **18-29 years** | 0.21752266 | 0.80060423 | 1.6858006 | 1.0392749 | 0.9123867 | 0.6132931 | 0.3444109 | 0.1389728 |  |
|  | **30-39 years** | 0.20689655 | 0.35344828 | 0.6831897 | 0.9396552 | 0.7306034 | 0.6810345 | 0.3685345 | 0.2219828 |  |
|  | **40-49 years** | 0.10489510 | 0.49883450 | 0.4522145 | 0.7179487 | 0.8205128 | 0.5058275 | 0.2843823 | 0.3006993 |  |
|  | **50-59 years** | 0.01486200 | 0.67091295 | 0.6496815 | 0.6772824 | 0.8152866 | 0.7834395 | 0.3651805 | 0.1974522 |  |
|  | **60-69 years** | 0.08033827 | 0.28118393 | 0.3678647 | 0.5983087 | 0.5073996 | 0.4799154 | 0.4883721 | 0.3023256 |  |
|  | **70+ years** | 0.04040404 | 0.09427609 | 0.2424242 | 0.2929293 | 0.2996633 | 0.1952862 | 0.2693603 | 0.2727273 |  |

**Table B16:** December 2020 – Home

|  |  | **Contact Age Group** | | | | | | | |
| --- | --- | --- | --- | --- | --- | --- | --- | --- | --- |
|  |  | **0-4 years** | **5-17 years** | **18-29 years** | **30-39 years** | **40-49 years** | **50-59 years** | **60-69 years** | **70+ years** |
| **Participant Age Group** | **0-4 years** | *NA* | *NA* | *NA* | *NA* | *NA* | *NA* | *NA* | *NA* |
|  | **5-17 years** | *NA* | *NA* | *NA* | *NA* | *NA* | *NA* | *NA* | *NA* |
|  | **18-29 years** | 0.062874251 | 0.08083832 | 0.27544910 | 0.09580838 | 0.07185629 | 0.06886228 | 0.03892216 | 0.008982036 |
|  | **30-39 years** | 0.051612903 | 0.05806452 | 0.04946237 | 0.13333333 | 0.05376344 | 0.04301075 | 0.04516129 | 0.015053763 |
|  | **40-49 years** | 0.030162413 | 0.09976798 | 0.06496520 | 0.05104408 | 0.12064965 | 0.04872390 | 0.03944316 | 0.041763341 |
|  | **50-59 years** | 0.006342495 | 0.05708245 | 0.10147992 | 0.05073996 | 0.06765328 | 0.13319239 | 0.06553911 | 0.038054968 |
|  | **60-69 years** | 0.006342495 | 0.02959831 | 0.05919662 | 0.06765328 | 0.02748414 | 0.07822410 | 0.17758985 | 0.063424947 |
|  | **70+ years** | 0.010101010 | 0.03367003 | 0.02356902 | 0.02693603 | 0.05723906 | 0.04377104 | 0.13468013 | 0.178451178 |

**Table B17:** December 2020 – Work

|  |  | **Contact Age Group** | | | | | | | | |
| --- | --- | --- | --- | --- | --- | --- | --- | --- | --- | --- |
|  |  | **0-4 years** | **5-17 years** | **18-29 years** | **30-39 years** | **40-49 years** | **50-59 years** | **60-69 years** | **70+ years** |  |
| **Participant Age Group** | **0-4 years** | *NA* | *NA* | *NA* | *NA* | *NA* | *NA* | *NA* | *NA* |  |
|  | **5-17 years** | *NA* | *NA* | *NA* | *NA* | *NA* | *NA* | *NA* | *NA* |  |
|  | **18-29 years** | 0.103244838 | 0.56932153 | 0.9380531 | 0.7758112 | 0.67256637 | 0.43362832 | 0.25073746 | 0.094395280 |  |
|  | **30-39 years** | 0.117521368 | 0.25427350 | 0.4871795 | 0.6282051 | 0.54273504 | 0.54059829 | 0.27136752 | 0.185897436 |  |
|  | **40-49 years** | 0.053117783 | 0.37413395 | 0.2886836 | 0.5519630 | 0.57505774 | 0.39953811 | 0.19399538 | 0.219399538 |  |
|  | **50-59 years** | 0.006342495 | 0.59408034 | 0.4101480 | 0.4989429 | 0.62579281 | 0.54545455 | 0.21775899 | 0.101479915 |  |
|  | **60-69 years** | 0.056722689 | 0.18487395 | 0.1932773 | 0.3256303 | 0.32352941 | 0.30252101 | 0.18067227 | 0.159663866 |  |
|  | **70+ years** | 0.023569024 | 0.03030303 | 0.1144781 | 0.1111111 | 0.07407407 | 0.04713805 | 0.03703704 | 0.006734007 |  |

**Table B18:** December 2020 – School

|  |  | **Contact Age Group** | | | | | | | |
| --- | --- | --- | --- | --- | --- | --- | --- | --- | --- |
|  |  | **0-4 years** | **5-17 years** | **18-29 years** | **30-39 years** | **40-49 years** | **50-59 years** | **60-69 years** | **70+ years** |
| **Participant Age Group** | **0-4 years** | *NA* | *NA* | *NA* | *NA* | *NA* | *NA* | *NA* | *NA* |
|  | **5-17 years** | *NA* | *NA* | *NA* | *NA* | *NA* | *NA* | *NA* | *NA* |
|  | **18-29 years** | 0.005882353 | 0.023529412 | 0.044117647 | 0.000000000 | 0.008823529 | 0.000000000 | 0.000000000 | 0.002941176 |
|  | **30-39 years** | 0.012793177 | 0.006396588 | 0.008528785 | 0.006396588 | 0.012793177 | 0.004264392 | 0.002132196 | 0.000000000 |
|  | **40-49 years** | 0.002309469 | 0.000000000 | 0.002309469 | 0.004618938 | 0.002309469 | 0.000000000 | 0.000000000 | 0.000000000 |
|  | **50-59 years** | 0.000000000 | 0.010548523 | 0.002109705 | 0.002109705 | 0.000000000 | 0.002109705 | 0.002109705 | 0.002109705 |
|  | **60-69 years** | 0.000000000 | 0.002100840 | 0.000000000 | 0.000000000 | 0.000000000 | 0.000000000 | 0.000000000 | 0.000000000 |
|  | **70+ years** | 0.000000000 | 0.000000000 | 0.000000000 | 0.003367003 | 0.000000000 | 0.000000000 | 0.000000000 | 0.000000000 |

**Table B19:** December 2020 – Other

|  |  | **Contact Age Group** | | | | | | | | |
| --- | --- | --- | --- | --- | --- | --- | --- | --- | --- | --- |
|  |  | **0-4 years** | **5-17 years** | **18-29 years** | **30-39 years** | **40-49 years** | **50-59 years** | **60-69 years** | **70+ years** |  |
| **Participant Age Group** | **0-4 years** | *NA* | *NA* | *NA* | *NA* | *NA* | *NA* | *NA* | *NA* |  |
|  | **5-17 years** | *NA* | *NA* | *NA* | *NA* | *NA* | *NA* | *NA* | *NA* |  |
|  | **18-29 years** | 0.053571429 | 0.125000000 | 0.4047619 | 0.1547619 | 0.1458333 | 0.10119048 | 0.05059524 | 0.03273810 |  |
|  | **30-39 years** | 0.023554604 | 0.032119914 | 0.1349036 | 0.1648822 | 0.1241970 | 0.08779443 | 0.04710921 | 0.01927195 |  |
|  | **40-49 years** | 0.018604651 | 0.027906977 | 0.1186047 | 0.1186047 | 0.1325581 | 0.06976744 | 0.04883721 | 0.04186047 |  |
|  | **50-59 years** | 0.002114165 | 0.006342495 | 0.1331924 | 0.1226216 | 0.1183932 | 0.09936575 | 0.07822410 | 0.05496829 |  |
|  | **60-69 years** | 0.016806723 | 0.063025210 | 0.1134454 | 0.2016807 | 0.1533613 | 0.09663866 | 0.12815126 | 0.07773109 |  |
|  | **70+ years** | 0.006734007 | 0.030303030 | 0.1043771 | 0.1515152 | 0.1683502 | 0.10437710 | 0.09764310 | 0.08754209 |  |
